# Case Report: Synchronous Manifestations of Kaposi Sarcoma Herpesvirus‐Associated Disorders

**DOI:** 10.1002/cnr2.70589

**Published:** 2026-06-01

**Authors:** Kandice Roberts, Paula Valiño Ramos, Saurabh Jindal, Vikas Prasad, Yi Shan Lee, Mariam Khanfar, Obi L. Griffith, Malachi Griffith, Jeanne Jiang, Ethel Cesarman, Daniel Rauch, Paul Simonson, Thomas Odeny, Lee Ratner

**Affiliations:** ^1^ Department of Medicine Washington University in St Louis School of Medicine St Louis Missouri USA; ^2^ Department of Microbiology Washington University in St Louis School of Medicine St Louis Missouri USA; ^3^ Department of Radiology Washington University in St Louis School of Medicine St Louis Missouri USA; ^4^ Department of Pathology Washington University in St Louis School of Medicine St Louis Missouri USA; ^5^ Department of Genetics Washington University in St Louis School of Medicine St Louis Missouri USA; ^6^ Department of Pathology and Laboratory Medicine Weill Cornell Medicine New York New York USA; ^7^ Department of Laboratory Medicine and Pathology University of Washington Seatle Washington USA

**Keywords:** castleman, daratumumab, effusion, herpesvirus, Kaposi, KSHV, lymphoma, sarcoma

## Abstract

**Background:**

Kaposi sarcoma herpes virus (KSHV) is associated with multiple clinical manifestations, including primary effusion lymphoma, an aggressive CD38+ B cell lymphoma with a plasmablastic phenotype.

**Case:**

This case describes an antiretroviral therapy‐adherent person with HIV who presented with concurrent KSHV‐related disorders of Kaposi sarcoma (KS), multicentric Castleman disease (MCD), and extracavitary primary effusion lymphoma (EC‐PEL). Single‐cell RNAseq (scRNAseq) and multiplex immunohistochemistry (mIHC) provide detailed insights into differences in cellular composition and viral and cellular transcriptomic differences between these diseases. Moreover, the case describes a long‐term remission with single‐agent anti‐CD38 antibody, daratumumab, in a chemotherapy‐refractory case.

**Conclusion:**

This case highlights the differing clinical manifestations of KSHV and the efficacy of immunotherapy in KSHV‐associated primary effusion lymphoma.

## 1 Background

In people living with HIV, there is increased risk of infection with oncoviruses and impairment of immunologic control of virus‐infected cells, permitting the development of cancer [1]. Kaposi's sarcoma‐associated herpes virus (KSHV), also known as human herpesvirus 8 (HHV‐8), is a gammaherpesvirus that has been associated with multiple malignancies in people with HIV, including Kaposi sarcoma (KS), multicentric Castleman disease (MCD), and primary effusion lymphoma (PEL) [1]. Patients may present with two or more of these diseases at the same time [1].

Since the advent of antiretroviral therapy (ART), there has been a significant decrease in the incidence of Kaposi sarcoma in the United States (US) [1], but there are still 1000–1400 new cases per year [2]. However, the incidence remains significantly higher among people with HIV compared to the general population, with a standardized incidence ratio of 214 from 2015 to 2019 [3]. Among those diagnosed with KS, those on ART with desirable CD4 counts and viral suppression are becoming increasingly represented [4]. In contrast, the incidence of MCD has been increasing over time from 2.3 per 10 000 patient years from 1983 to 1996 to 8.3 per 10 000 patient years from 2002 to 2007, according to a European study of a prospective HIV database [5]. The epidemiology of PEL has been more difficult to characterize, given that it is exceedingly rare [6]. A retrospective cohort study in the US showed a greater than 700‐fold higher incidence of PEL among those living with HIV compared to the general population from 2001 to 2019 [7]. PEL likely remains underdiagnosed, especially in regions with high seroprevalence of HIV such as sub‐Saharan Africa, due to its unusual presentations [6].

Primary effusion lymphoma is a rare clonal B‐cell lymphoma that typically presents as a body cavity effusion but can also manifest as extracavitary disease [1]. KSHV infects tumor cells in all cases of primary effusion lymphoma, and co‐infection with Epstein–Barr virus (EBV) is found in up to 90% of cases [8]. Extracavitary or solid variants of PEL often lack expression of B‐cell antigens and are commonly co‐infected with EBV [8]. However, they have the morphology of large cell immunoblastic or plasmablastic lymphomas [8]. Classically, primary effusion lymphoma (PEL) has been treated with combination chemotherapy regimens used to treat other aggressive B‐cell lymphomas, as well as antiretroviral therapy [1]. However, long‐term remission with this approach is only achieved in approximately 40% of patients [1]. Despite multiple recent advances in the treatment of HIV‐associated malignancies, PEL still carries a relatively poor prognosis in people with HIV [9]. Improved therapies are urgently needed to address this disparity.

There has been a recent interest in the use of daratumumab for the treatment of PEL. Daratumumab is a humanized IgGκ monoclonal antibody that targets CD38, a transmembrane glycoprotein that is highly expressed on malignant blood cells [10]. It was initially approved in the US in 2015 for use in patients with multiple myeloma who had disease progression after at least three prior lines of therapy [10]. It has been shown to exert its anti‐myeloma effect via direct cytotoxicity, immune‐mediated tumor cell lysis, and immunomodulatory effects [10]. Since its initial approval, its use has expanded to frontline treatment of multiple myeloma, high‐risk smoldering multiple myeloma, and light chain amyloidosis [11]. Efficacy of daratumumab for PEL has been supported by preclinical studies. One study of PEL cell samples from 8 patients revealed high levels of CD38 expression by flow cytometry [12]. When these cells were treated with daratumumab, there was significant dose‐dependent induction of antibody‐dependent cell‐mediated cytotoxicity [12]. Daratumumab has also been shown to induce NK‐cell‐mediated cytotoxicity and complement‐dependent cytolysis in PEL cells and inhibit PEL tumor growth in a xenograft model [13]. Daratumumab has been administered in two cases of PEL before, achieving clinical response [12, 16].

We report a patient presenting with synchronous KS, MCD, and progressive, chemotherapy‐refractory extracavitary PEL (EC‐PEL). His PEL achieved a complete metabolic response on PET‐CT imaging following 8 weeks of treatment with single‐agent daratumumab. He maintained remission on daratumumab for more than 2 years.

## 2 Case

In November 2022, a 43‐year‐old man with a history of well‐controlled asthma presented to Barnes Jewish Hospital in St. Louis, MO with several weeks of night sweats, fatigue, and intermittent fevers. A CT of the chest, abdomen, and pelvis showed prominent bilateral axillary, mediastinal, and hilar lymphadenopathy as well as right apical pleural blebs. Further workup was notable for positive HIV 4th generation testing. The HIV viral load was 553 000 copies/mL, and his CD4 count was 105 cells/mcL. Due to his extensive lymphadenopathy, he underwent a broad infectious workup including bartonella, tularemia, toxoplasmosis, tuberculosis, cryptococcus, blastomycosis, histoplasmosis, salmonella, and acid‐fast bacilli testing, all of which were negative. He also underwent a right inguinal excisional lymph node biopsy, and pathology was consistent with reactive lymphoid tissue. Staining for microorganisms was negative, and there was no sign of malignancy. The infectious diseases (ID) team was consulted and recommended starting Biktarvy daily. He was also found to have glucose‐6‐phosphate dehydrogenase (G6PD) deficiency during admission, so he was started on atovaquone for possible Pneumocystis jirovecii pneumonia (PJP) given his chest imaging findings of apical blebs. He experienced symptom improvement during admission and was discharged with outpatient ID follow up.

His HIV viral load improved to 311 copies/mL by December 2022. Then, at a follow up visit in January 2023, he was noted to have approximately 12 new slightly raised, hyperpigmented, violaceous lesions on his arms and legs. His CD4 count at that time had also fallen to 32 cells/mcL despite reported complete adherence to antiretroviral therapy. He was referred to dermatology for a biopsy of a skin lesion, and an appointment was scheduled for March 2023. By February 2023, he had developed new subcutaneous nodules on his trunk and abdomen, a large nodule on his back, a painful nodule on the inner aspect of his right nostril with occasional associated white drainage and nasal obstruction, nodules in the bilateral pre‐auricular and submandibular areas, right sided headaches, fatigue, dyspnea on exertion, and intermittent fevers to 103°F. He was instructed to present to the hospital for further workup and management.

On admission, he was found to be tachycardic to 116 beats per minute with a temperature of 101.6°F. Physical exam was notable for widespread hyperpigmented plaques with slightly raised edges on his bilateral upper and lower extremities, scattered firm subcutaneous nodules with the most pronounced measuring 2–3 cm in diameter between his scapulae on his upper back, and a nodular pinkish lesion with some overlying vessels in the right nares. His lungs were clear to auscultation bilaterally, and there was no clear palpable lymphadenopathy. His CD4 count on admission was < 35 cells/mcL, and his HIV viral load was again undetectable. An infectious workup was negative for mycobacteria, respiratory viruses, blastomycosis, ehrlichiosis, *Bartonella henselae* , *Bartonella quintana* , histoplasmosis, anaplasmosis, and hepatitis A, B, and C. Both bacterial and fungal blood cultures were negative. A CT scan of the chest, abdomen, and pelvis showed persistent generalized lymphadenopathy which was mostly unchanged compared to his scan from November 2022 and some small ground glass nodules in the bilateral upper lung lobes. Histopathology from a biopsy of a hyperpigmented skin patch on the right forearm as well as positive human herpes virus 8 (HHV‐8, KSHV) staining confirmed Kaposi sarcoma (Figure 1A,B). A biopsy of the largest nodular skin lesion on the back was diffusely and strongly positive for CD45, CD3, MUM‐1, EBV, and KSHV, which was most compatible with extracavitary primary effusion lymphoma (Figure 1H). He also had a biopsy of a right nasal lesion which was positive for HHV‐8 and reported as a malignancy consistent with involvement by EC‐PEL, arising in the spectrum of MCD (Figure 1E–G). Fluorescence in situ hybridization of the nasal cavity lesion was negative for *MYC* gene rearrangements. Quantitative reverse transcription polymerase chain reaction (RT‐PCR) testing of whole blood for HHV‐8 DNA revealed a viral load of 2 337 271 copies/mL (Figure S1). Histopathology and flow cytometry from a bone marrow biopsy were negative for lymphoma. PET‐CT showed an FDG‐avid right nasal passage lesion, numerous lymph nodes, and subcutaneous lesions (Figure 2A,D) and absence of any intracardiac lesion (Figure 2E). He underwent an excisional biopsy of a right jugular lymph node, which was morphologically most consistent with MCD with focal involvement of EC‐PEL within sinuses (Figure 1C,D). Flow cytometry of the right jugular lymph node tissue detected no clonal B‐cell or aberrant T‐cell populations. Cerebrospinal fluid cytology was negative for malignancy, and flow cytometry was not performed due to insufficient cells.

**FIGURE 1:**
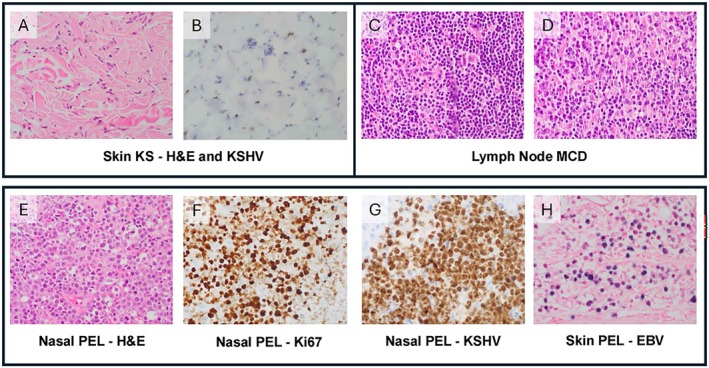
Pathology Studies. (A) A hematoxylin and eosin (H&E) stain of KS lesion tissue from a punch biopsy of the patient's forearm showing fascicles of spindle cells. (B) Weak HHV‐8 latency‐associated antigen positivity in a portion of lesional cells, confirming a diagnosis of Kaposi sarcoma. (C) An H&E stain of right jugular lymph node tissue which reveals the presence of secondary follicles with prominent mantle zones. (D) An H&E stain of right jugular lymph node tissue which shows interfollicular areas expanded by sheets of mature‐appearing plasmacytoid cells admixed with scattered larger cells reminiscent of plasmablasts. (E) An H&E stain of fragments of squamous mucosa with ulceration and a dense submucosal infiltrate of large atypical mononuclear cells with high nuclear to cytoplasmic ratio, moderate eosinophilic cytoplasm, round nuclei, and prominent nucleoli. Increased background mitoses, apoptosis, and focal areas of necrosis are also seen. (F) An immunohistochemical (IHC) stain of nasal lesion tumor cells demonstrating a Ki67 proliferation index of approximately 80%. (G) IHC staining of nasal lesion tissue for KSHV (HHV‐8), which is strongly positive. (H) Tissue from a skin punch biopsy of the patient's back demonstrates significant positivity on Epstein Barr virus encoded RNA (EBER) in situ hybridization staining. All panels show images taken at 60× magnification with a bar for 20 μM shown in the lower right corner of each micrograph.

**FIGURE 2:**
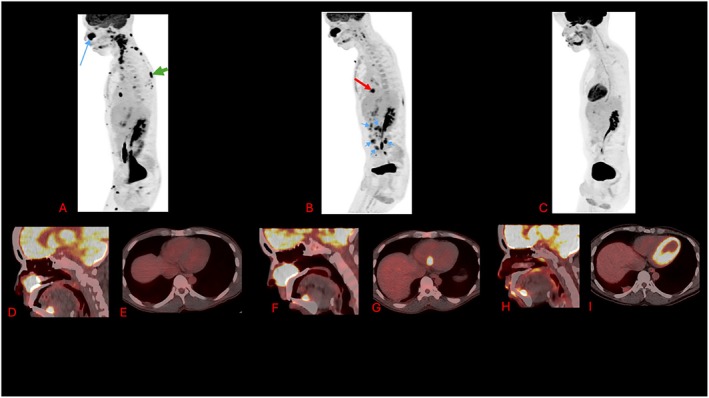
PET/CT studies. Images A, B, and C show maximum intensity projections of F‐18 fluorodeoxyglucose (FDG) positron emission tomography (PET) scan in sagittal view from the time of diagnosis (03/02/2023), disease progression (09/25/2023), and remission (12/08/2023), respectively. Image A shows anterior nasal cavity lesion (target lesion, blue arrow), cutaneous lesion on the back (histopathology positive for HHV‐8 and EBV, green arrow), multiple neck lymph nodes and cutaneous nodules. Image B shows increase in the size of the target lesion, new intracardiac focus (red arrow), and multiple new intramural foci in the small bowel (blue arrows), consistent with disease progression. Image C shows complete anatomic and metabolic resolution of the targeted and other metastatic lesions consistent with treatment response. Images D, F and H show fused PET‐CT images at the level of the nasal lesion in sagittal view, whereas G is showing fused axial image of intracardiac lesion that was absent on first PET (E) but resolved after daratumumab therapy (I). The maximum standard uptake value of the target lesion was 15.3 at the time of diagnosis.

From March to June 2023, he received six cycles of dose adjusted etoposide, prednisone, vincristine, cyclophosphamide, doxorubicin, and rituximab (DA‐R‐EPOCH) with intrathecal chemotherapy for EC‐PEL. Intrathecal chemotherapy was prophylactic in this case, given the aggressive nature of EC‐PEL. Treatment with this regimen was complicated by grade 3 febrile neutropenia, grade 3 thrombocytopenia, and grade 1 peripheral sensory neuropathy by Common Terminology Criteria for Adverse Events, Version 6 (CTCAE v6). His CD4 count dropped to < 35 cells/mcL following initiation of chemotherapy and remained undetectable throughout treatment with this regimen. A PET‐CT in August 2023 demonstrated refractory disease. He received one cycle of salvage therapy with gemcitabine and oxaliplatin in August 2023, which was complicated by grade 3 thrombocytopenia by CTCAE v6, and he experienced subsequent progressive disease in the right nares, cervical lymph nodes, heart, cecum, and peritoneum (Figure 2B,F,G). The patient's treatment was therefore switched to daratumumab in September 2023. At the start of this treatment regimen, his CD4 count was 42 cells/mcL. Approximately 6 h after his first daratumumab infusion, he experienced rigors and a fever to 103 degrees Fahrenheit. He was treated with 50 mg diphenhydramine, 40 mg famotidine, and 25 mg meperidine and experienced symptom and fever resolution within 3 h. Blood cultures were drawn and were negative. He continued to receive daratumumab once weekly from weeks 1 to 8, with the addition of oral dexamethasone 20 mg to each dose, and he experienced no further infusion reactions. Following his 7th infusion of daratumumab, he was diagnosed with a pulmonary embolism and asymptomatic COVID‐19 infection, requiring hospitalization. He subsequently recovered and was discharged on oral anticoagulation. Of note, his whole blood KSHV DNA viral load from 11/01/2023 was 500 copies/mL, which was decreased from 330 020 on 10/04/2023 (Figure S1). PET CT scan in December 2023 demonstrated a complete response to treatment (Figure 2C), including the nasal passage lesion (Figure 2H) and intracardiac lesion (Figure 2I). Thus, daratumumab was administered once every 2 weeks from weeks 9 to 24 and once every 4 weeks thereafter as maintenance. He completed 2 years of therapy on daratumumab, then received four additional monthly treatments after shared decision making. His last treatment was January 2026. He has remained in remission. His KSHV viral load was undetectable on 12/26/2024 (Figure S1). His CD4 count also gradually improved throughout treatment with daratumumab and was 325 cells/mcL on 2/25/2026.

## 3 Single‐Cell RNA Sequencing Analyses

### 3.1 Kaposi Sarcoma Lesion Reveals a Typical KS Transcriptional Landscape

A Kaposi Sarcoma lesion on the right thigh was biopsied in March 2023 and submitted for single‐cell RNA sequencing (scRNAseq). The lesion was compared to a recently published report of single‐cell transcriptomics from 19 KS lesions from the Midwest, USA [14]. Figure 3 shows the cell type distribution (Figure 3A), sample distribution (Figure 3B), and CD38 expression (Figure 3C) in the composite object of these KS lesions. Transcriptomic characteristics of our case's lesion, designated KS14, were comparable to this dataset in terms of tumor and immune cell composition and distribution (Figure 3A,B). About 1% of the KS14 tumor cells exhibited more than 1 KSHV transcript, consistent with the level of KSHV detection observed in scRNAseq of other KS lesions (Figure S2A) [14]. As previously reported, the KSHV+ cells were found to be lymphatic endothelial cells (Figure 3A) [14].

**FIGURE 3:**
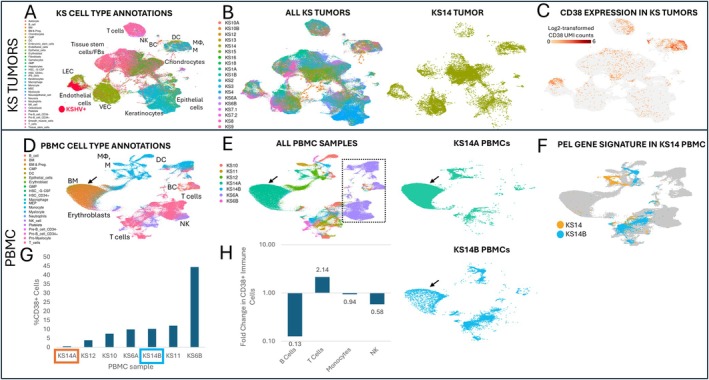
Single cell RNA sequencing (scRNAseq) of Kaposi Sarcoma and PBMC. Panels A–C show a composite UMAP of a KS skin lesion from the case subject with 18 published lesions from non‐PEL KS patients [14]. (A) Cell identity annotations in the KS composite object assigned by SingleR. KSHV+ cells have been highlighted in red. > 95% of KSHV+ cells reside in a single endothelial cell cluster, with < 5% scattered in other clusters across multiple cell types. (B) Distribution of cells from each tumor in the composite object. The green panel separately shows the cells from KS14. (C) Expression of CD38 in the composite object. The orange color gradient reflects detection of CD38 unique molecular identifiers (UMIs) in logarithmic scale. Panels D–F show a composite UMAP of two longitudinal PBMC samples from the case subject with 5 published PBMC samples from non‐PEL KS patients [14]. (D) Cell identity annotations in the PBMC composite object assigned by SingleR. (E) Distribution of cells from each PBMC sample in the composite object. The green and light blue panels separately show the cells from the initial PEL presentation timepoint (KS14A) and the complete remission sample (KS14B), respectively. (F) Cells from KS14A (orange) and KS14B (light blue) PBMC that exhibit a known PEL gene signature: CD20−, CD19−, CD79a−, PAX5− [15]; and > 4 transcripts of CD45. (G) Percentage of total CD38+ cells in each PBMC sample. CD38+ cells were included by featuring at least 1 CD38 transcript. (H) Fold change in CD38+ B cells, T cells, monocytes, and NK cells between the baseline PEL and complete remission samples. The change is presented in logarithmic scale. Legend: BC = B cells; BM = bone marrow‐derived cells; DC = dendritic cells; FBs = fibroblasts; LEC = lymphatic endothelial cells; MΦ = macrophages; M = monocytes; NK = natural killer cells; UMI = unique molecular identifier; VEC = vascular endothelial cells. Kaposi Sarcoma skin lesions were collected via punch biopsy and dissociated to a single cell suspension using a Whole Skin Dissociation Kit (Miltenyi Biotech). Cells were viably frozen in Bambanker serum‐free cell freezing media (Sigma) at −80°C and thawed immediately prior to submission for scRNAseq. Approximately 50 000 cells per sample were submitted. For PBMC preparation, whole blood was centrifuged to separate plasma from the cellular fraction, and white blood cells were separated from RBCs via ficoll density gradient. RBC contamination was cleaned with Red Blood Cell Lysis Buffer (Roche). Cells were viably frozen as described above. Around 20 000 cells per sample were submitted for scRNAseq. Library preparation was performed at GTAC@MGI with the 10× Genomics 5′ or 3′ immune profiling kits. Libraries were sequenced on Illumina S4 flow cells (300 cycles, 100 000 reads/cell). CellRanger multi pipeline (v9.0.1) was used for alignment and gene expression. Reads were mapped against the human (GRCh38/hg38) and KSHV (Strain GK18, RefSeq Organism 37 296, ViralProj14158, GCF_000838265.1) genomes. The feature‐barcode matrices were processed with Seurat (v5.3.0) using the default settings. Data was visualized using uniform manifold approximation and projection (UMAP) plots. Cells were annotated with SingleR (v2) using expression profiles from the Human Primary Cell Atlas (HPCA) dataset. Differential gene expression and visualization were performed using Loupe Browser 9 (10× Genomics).

While low CD38 transcripts were scattered in most clusters, the highest CD38 expression was concentrated in immune cell clusters, as well as a KSHV‐negative endothelial cell subcluster (Figure 3C). The highest CD38 unique molecular identifier (UMI) counts were found in macrophages, which made up 19% of CD38+ cells in tumors, and KSHV− endothelial cells, which comprised 17%. Low CD38 transcripts were scattered in most cell types (Figure 3A,C). CD38+ cells were a minority in each of the tumors, making up less than 3% of the cells in all but one participant's lesion (Figure S2B). In the KS14 lesion, 1.4% of the cells had detectable CD38 (Figure S2B), which was scattered in multiple cell types as described above. There were more CD38+ monocytes than any other immune cell types in this sample, and 60% of CD38+ cells were endothelial cells, fibroblasts, tissue stem cells, or smooth muscle cells. No CD38 was detected in B cells or plasma cells in KS14.

### 3.2 Peripheral Blood Mononuclear Cells in PEL Patient Compared to Non‐PEL KS Patients

Peripheral blood mononuclear cells (PBMC) were collected near the initial presentation with PEL, in March 2023, and again 8 months after the patient achieved complete remission, in July 2024. These samples were submitted for scRNAseq and compared to five published samples of PBMC from KS patients without PEL [14]. KSHV transcripts were absent from any of the PBMC samples using this method, consistent with the previous description of this dataset. Most PBMC samples from KS patients without PEL showed a similar cellular composition but did not merge as well as the KS lesions (Figure 3D,E). One PBMC sample collected prior to ART initiation in one subject, KS6A, migrated distinctly from the rest of the other PBMC samples, clustering separately (Figure 3E, purple cells in box). PBMC from the same patient, 6 months after ART was started, KS6B, merged and clustered with the rest of the PBMC samples (Figure 3E, pink cells). KSHV genomes from patient KS6 were found to have an amplification in the K5‐K7 region [14], however, no KSHV transcripts were detected in PBMC from KS6A or any other PBMC samples.

The PBMC of the PEL patient introduced a new cell cluster absent in the non‐PEL dataset (Figure 3D,E, arrow). This cluster migrated closely with monocytes from the PEL initial presentation, and it was composed almost exclusively of the PEL patient's cells, with a small contribution from one non‐PEL patient, KS12. Differential gene expression of this cluster versus the rest of the dataset revealed upregulation of hemoglobin chains and heme synthesis pathway actors. This led to SingleR classification of these cells as a mixture of bone marrow‐derived, nucleated red blood cell precursors (erythroblasts), and hematopoietic stem cells. A small proportion of baseline PEL cells in this cluster were classified as pro‐myelocytes. Given the lack of clinical evidence indicating a neoplasm of myeloid lineage and the patient's chronic anemia in the context of G6PD deficiency, the presence of high red blood cell (RBC) precursors in the peripheral blood was likely a manifestation of the patient's chronic anemia. At the complete remission timepoint, the patient's hemoglobin and hematocrit were higher than at the initial presentation, yet still below the normal range. In concordance with this, we found fewer erythroid precursors in the complete remission sample than the baseline PEL sample. At the time of PBMC collection, patient KS12 had low hematocrit, RBCs, and hemoglobin. This may be related to the small presence of erythroid precursors in this sample.

### 3.3 No Evidence of Malignant Cells in PBMC

While blood findings, such as CD4+ lymphocyte depletion, can accompany the presentation of PEL [5], the neoplastic cells are commonly identified in effusions, or nodal or extracavitary masses [15]. Consistent with these observations, we found no evidence of malignancy in the peripheral blood. The small proportion of B cells in the baseline PBMC PEL sample was similar to that found in the complete remission sample, and it was also comparable to B cell frequency in PBMC from KS patients without PEL. Although PEL malignant cells are known to be of B cell origin [15], classical B cell markers CD19 and CD20 are notably absent in PEL cells [15]. Further, all PEL neoplasms are KSHV+, and over 90% are also EBV+ [8]. KSHV was detected in the nasal and cutaneous PEL lesions, but no significant KSHV transcripts were found in the baseline PBMC despite a viral load of over 2 000 000 copies/mL (Figure S1). This further supports the lack of PEL neoplastic cells in the peripheral blood. PEL cells sometimes express immunoglobulin light chains, but the baseline PEL PBMC exhibited no clonal B cell expansion (Figure S3A).

The only marker known to be consistently expressed in PEL cells, CD45 (PTPRC), appeared to be uniquely downregulated at the transcriptional level in the baseline PEL PBMC compared to the complete remission PBMC sample and to the non‐PEL samples. However, this difference was found to be mediated by the erythroid precursor cells in the baseline PEL PBMC (Figure S3B). In cells that featured the PEL gene signature (CD20−, CD19−, CD79a−, PAX5−) [15], CD45 was expressed at low levels (1–2 transcripts per cell) in a relatively small subset of the erythroid precursor cluster (19% of the cluster). The rest of the erythroid precursor cluster was either CD45− (79%) or did not feature the PEL gene signature (1.6%). Cells that featured the PEL immunophenotypic signature and expressed higher levels of CD45 transcripts (> 4) included monocytes and T cells and, to a lesser extent, NK cells and granulocyte colony‐stimulating factor hematopoietic stem cells (G‐CSF HSC) (Figure S3C). However, PEL cells may aberrantly express T cell markers, and they are always KSHV+ [15], which was not found in this cell population. In addition, the complete remission sample featured the same expression pattern in monocytes and T cells, further discouraging the identification of these populations as malignant (Figure 3F). Furthermore, no cell population that expressed PEL markers was present in the baseline sample and absent in the complete remission sample, or vice versa. Together, these observations suggest the absence of peripheral blood involvement by PEL in this patient.

### 3.4 CD38 Transcription Increases in PBMC After Daratumumab Treatment

An unexpected finding was the increased detection of CD38+ cells in PBMC during complete remission compared to the initial presentation (Figure 3G). Daratumumab is expected to target CD38+ cells. Notably, the percentage of CD38 RNA expressing cells at the PEL baseline was lower than in all other samples. The proportion of CD38+ cells increased to over 10% of total detected PBMC at complete remission (Figure 3G), which was similar to the non‐PEL PBMC. In KS14B, CD38 RNA expression was found mostly in T cells, monocytes, and natural killer cells, which together comprised 97% of the CD38+ cells. To evaluate whether the large proportion of erythroblast precursors in the KS14 samples impacted these proportions, the calculations were repeated excluding erythroblasts, bone marrow‐derived cells, and pro‐myelocytes from the KS14A and KS14B cell counts. The proportion of CD38+ cells mildly increased from 0.6% to 2.5% in KS14A, and from 10.2% to 11.7% in KS14B; however, the observed trend remained the same (Figure 3G and Figure S3D). This peripheral blood finding could suggest the recovery of normal immune populations after lymphoma remission. However, as protein levels were not evaluated in this study, the effect of daratumumab in these peripheral blood cells cannot be fully ascertained. Interestingly, expression of CD38 in the non‐PEL longitudinal samples (KS6A and KS6B) also increased after ART initiation (Figure 3G). KS6B had the highest proportion of CD38+ cells among all PBMC samples. This suggests that an increase in CD38‐expressing immune cells correlates with immune system recovery.

### 3.5 Daratumumab Increases CD38+ T Cells, Decreases CD38+ B Cells in Peripheral Blood

To further understand the changes in CD38 expression, we evaluated the distribution of CD38 among different immune cell types in the initial PEL presentation compared to the complete remission PBMC. We excluded all cells classified as erythroblasts, bone marrow‐derived, and pro‐myelocytes from this analysis. Compared to the initial presentation PBMC, there was a 2.1‐fold increase in CD38+ T cells at complete remission (Figure 3H). CD38+ T cells were 55% of the total CD38+ PBMC present during daratumumab maintenance, suggesting either a lack of CD38 protein expression in these cells or protection from daratumumab targeting. On the other hand, the opposite change was observed in CD38+ B cells. Despite a low proportion of B cells in both longitudinal PBMC samples, there was a nearly 8‐fold decrease in CD38+ B cells at complete remission versus initial presentation (Figure 3H). This could indicate that daratumumab can deplete the CD38‐expressing B cell population in the peripheral blood. There was also a 1.7‐fold decrease in CD38+ NK cells at complete remission compared to the initial presentation, and monocytes remained unchanged (Figure 3H).

## 4 Multiplex Immunofluorescence

The KS skin biopsy (Figure 4A,B), one lymph node with PEL involvement (Figure 4C,D), one lymph node with MCD involvement (Figure 4E,F), and a control tonsil sample (Figure 4G,H) were submitted for multiplex spatial proteomics using the Lunaphore COMET system. In the KS lesion, LANA+ cells were found in the endothelial cells of some vessel structures (Figure 4A,B red arrows) and scattered throughout the dermis (Figure 4B, black arrows). CD38 and CD138 protein expression were ascribed to plasma cells. There was comparatively little CD38 signal in other cells, and some low‐level CD138 expression was ascribed to epithelial cells (Figure 4A).

**FIGURE 4:**
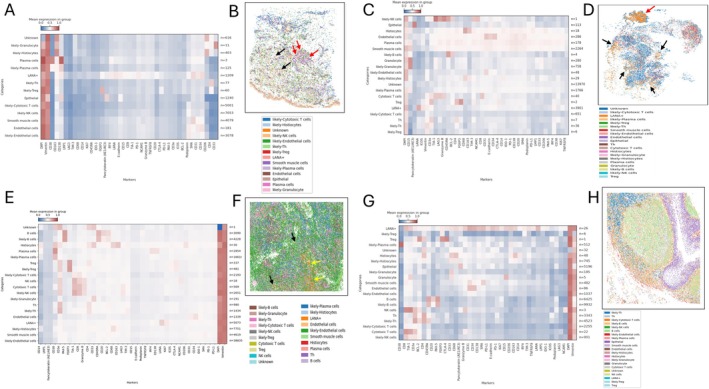
Multiplex immunofluorescence study of KSHV‐associated disorders. The mean expression heat maps and spatial distribution of cell type assignments (supervised clustering) are shown in (A, B) Kaposi Sarcoma, (C, D) a lymph node with PEL involvement, (E, F) lymph node with MCD involvement, and (G, H) control uninvolved tonsil tissue. The formalin‐fixed, paraffin‐embedded tissue samples from the patient and a control tonsil were used. Spatial imaging was performed using the Lunaphore COMET platform (Tholochenaz, Switzerland). The imaging area was 12.5 mm × 12.5 mm. Sequential immunofluorescence was performed, comprising iterative cycles of priming, antibody incubation, elution, quenching, counterstaining, and imaging at 37°C. Each imaging step captured three channels (DAPI, TRITC, Cy5) with exposure times of 25 ms (DAPI), 350–850 ms (TRITC), and 300–600 ms (Cy5). Washing employed Lunaphore Multistaining Buffer; elution used Lunaphore Elution Buffer at 37°C. Primary antibodies were applied in paired cycles (16 h incubation), followed by secondary antibodies (anti‐mouse AF555, anti‐rabbit AF647) for 2 min. The panel targeted viral, immune, and stromal markers, including LMP1, LANA, CD3e, CD4, CD8, CD15, CD20, CD31, CD33, CD38, CD45RA, CD68, CD138, CD163, ICOS, FOXP3, PD‐1, PD‐L1, TIM‐3, LAG‐3, IDO‐1, CTLA‐4, Ki67, SMA, NCAM1, vimentin, E‐cadherin, BCL‐2, TIA‐1, TNFRSF8 (CD30), PAX5, podoplanin, pancytokeratin (AE1/AE3), and CD14. Raw images were processed in Lunaphore Horizon Viewer for background subtraction and channel selection. Multi‐channel TIFF files were exported for analysis. Cell segmentation was performed using Cellpose on a Ubuntu workstation with GPU acceleration. Segmentation masks were converted to tabular format using a custom python script, making use of scikit‐image and pandas python libraries. Per‐cell fluorescence intensities were extracted and normalized. Immunophenotyping and spatial analysis were conducted using scimap in Jupyter notebooks. Marker positivity thresholds were manually determined using visualization with napari. Cell populations were classified according to a predefined gating strategy (Supporting Information), encompassing major T cell subsets (CD3+ T cells, cytotoxic T cells, helper T cells, regulatory T cells, NK cells), B cells, plasma cells, histiocytes, granulocytes, vascular cells, and KS cells, as well as functional states (PD‐1^+^, TIM‐3^+^, LAG‐3^+^, ICOS^+^, IDO‐1^+^). Channels with poor signal were excluded.

In the sample involved by PEL (Figure 4C,D), part of the LANA+ cells clustered together (Figure 4D, red arrow), while another fraction was scattered around a large cluster of cells of unidentified origin (Figure 4D, black arrows). CD38 was most highly expressed in plasma cells, but low‐level expression was also present in T cells, LANA+ cells, smooth muscle cells, endothelial cells, and histiocytes. This was consistent with the scattered CD38 transcription found in the scRNAseq data, which was particularly concentrated in T cells and B cells.

The lymph node with MCD involvement (Figure 4E,F) featured clustered LANA+ cells (Figure 4F, yellow arrows) along with scattered cells (Figure 4F, black arrows). CD38 was most highly expressed in plasma cells and histiocytes, and likely low‐level expression could also be found in T cells, B cells, and, to a lesser extent, in LANA+ cells (Figure 4E).

In the tonsil control tissue (Figure 4G,H), very few LANA+ cells were identified, and upon closer inspection, these were seen to be a result of non‐specific staining. The tonsil tissue demonstrated a normal lymphoid follicular architecture with overlying epithelium and essentially normal distribution of immune cells. CD38 and CD138 expression was seen in plasma cells and to a much lesser extent in other cells, including histiocytes; CD138 expression was also seen in epithelial cells, as expected.

## 5 Discussion

Our case demonstrates the efficacy of daratumumab in the treatment of extracavitary PEL, which is consistent with the available literature. There is both in vivo and in vitro evidence that daratumumab is active in PEL, given that CD38 is widely expressed in the majority of PEL cells. Daratumumab induces antibody‐dependent cellular cytotoxicity‐mediated lysis of PEL cell lines and was shown to induce a clinical response in two cases of refractory PEL with leptomeningeal involvement at the National Cancer Institute [12]. Previously, two other cases of PEL were treated with daratumumab with clinical response, including one case with heavily pre‐treated multiple refractory disease in a person with HIV and another in previously untreated PEL with pericardial involvement in a person without HIV [16, 17]. Importantly, in both cases, the KSHV DNA viral load levels decreased with treatment. Although KSHV viral load is not validated for monitoring disease response, it also trended downward in our patient as his PEL improved on daratumumab.

Coinfection with KSHV and HIV is common, due to their shared risk factors [18]. Moreover, KSHV‐associated disorders are much more common among people living with HIV [19]. In immunocompetent hosts, latent KSHV infection of B cells is controlled by KSHV‐specific CD4+ effector T cells and cytotoxic T lymphocytes [20, 21]. Both responses are lost as HIV‐driven immunodeficiency worsens. Our patient was able to achieve an undetectable HIV load following the initiation of ART but simultaneously experienced a fall in his CD4 count. This can likely be explained by the development of PEL, which can suppress CD4 counts via cytokine release by tumor cells [22]. Additionally, KSHV itself leads to direct immunosuppression via stimulation of monocyte‐derived dendritic cells and resultant impairment of resting CD4+ T cell proliferation [23]. EBV co‐infection, which was noted in this case, adds additional immunosuppression via EBV‐encoded latent membrane protein 1, which directly inhibits T‐cell activation and proliferation [24]. Our patient's CD4 count was then further suppressed following initiation of DA‐R‐EPOCH. This is a known side effect in people with HIV who receive this chemotherapy regimen [25].

Following initiation of daratumumab, our patient's CD4 count finally began to improve. Daratumumab is known to be a common cause of lymphopenia among patients undergoing treatment for multiple myeloma due to depletion of CD38‐high lymphoid populations [26]. However, this prompts a robust expansion of both CD4+ and CD8+ T cell populations, as seen in this case [27]. This paradoxical increase in CD4+ T cells may be of particular interest in people living with HIV. There are ongoing efforts to better understand the efficacy of daratumumab in the treatment of PEL. An active phase 2 trial sponsored by the National Cancer Institute (NCT05907759) is evaluating subcutaneous daratumumab for the treatment of relapsed/refractory PEL, plasmablastic lymphoma (PBL), and KSHV‐associated multicentric Castleman disease (MCD).

Additionally, the patient's KS resolved during the course of daratumumab therapy. Immune reconstitution alone is sufficient to manage KS in many people living with HIV [28]. As this patient was ART compliant at disease onset, the remission of his KS is likely associated with his PEL resolution and CD4 count improvement rather than a direct effect of daratumumab therapy.

Our study is novel in including transcriptomics and multiplex proteomics analyses of the patient's KSHV‐associated diseases. Single‐cell RNA sequencing analyses of our patient's KS lesion revealed no unique differences in cellular composition or KSHV‐infected cell count compared to other KS patients from the same geographical region in our prior report [14]. There was no peripheral blood involvement by PEL at initial presentation, which was concordant with the patient's clinical presentation. Multiplex immunofluorescence showed KSHV infection of KS endothelial cells, and of CD38+ cells in PEL and MCD. CD38+ plasma cells were identified in all samples, supporting the multi‐system daratumumab response in this patient and the availability of CD38 as a therapeutic target.

Our case's peripheral blood scRNAseq was notable for a high number of erythroblasts and hematopoietic stem cells, best explained by his chronic anemia in the context of G6PD deficiency. The absence of these precursor cells in other samples from patients on ART suggests they are unlikely to be related to ART effects. Our patient had a clinical history of well‐controlled asthma, not significant during his EC‐PEL course. G6PD deficiency, which impacts inflammasome function [29], may have predisposed our patient to autoimmunity.

An interesting finding was the overall increase in CD38+ PBMC in the complete remission sample compared to the baseline sample. The proportion of CD38+ cells in the complete remission sample, obtained during daratumumab maintenance, was similar to that of the non‐PEL samples. This suggests that higher CD38+ cell counts could be related to immune system recovery after PEL. We found that the CD38+ cell increase was largely driven by CD38+ T cells, while CD38+ B cells decreased 8‐fold. CD38 is known to be expressed at low levels in T cells, and increased CD38 expression is a marker of T cell activation [30, 31]. One study reported that daratumumab depleted highly CD38+ regulatory T cells, and it induced expansion of helper and cytotoxic T cells expressing lower levels of CD38 [27]. Another study showed that low CD38 expression diminishes daratumumab effector mechanisms, yielding inefficient cell depletion [32]. Low CD38 expression in our patient's circulating T cells might have led to relative daratumumab sparing compared to the efficient depletion of lymphoma and B cells, allowing recovery of his T cell counts. Our observation that daratumumab depleted CD38+ NK cells but not CD38+ monocytes is also consistent with other studies [33]. The interpretation of these results is limited by the lack of protein data.

## 6 Conclusion

Pathology, IHC, and highly multiplexed immunofluorescence analyses of the current case highlight the contrasting features of KSHV‐associated disease. Single‐cell RNAseq analysis of the KS skin lesion demonstrated typical features seen in other cases of KS. Single‐cell RNAseq analyses of the PBMC showed changes in the cellular transcriptome, including CD38 expression, seen as the patient responded to daratumumab therapy. This case demonstrates the efficacy of daratumumab for the treatment of relapsed/refractory EC‐PEL.

## Author Contributions

**Daniel Rauch:** writing – review and editing, supervision, visualization, formal analysis, data curation. **Paula Valiño Ramos:** writing – original draft, writing – review and editing, formal analysis, data curation, visualization. **Kandice Roberts:** writing – original draft, writing – review and editing, visualization. **Saurabh Jindal:** writing – review and editing, visualization, supervision. **Yi Shan Lee:** writing – review and editing, visualization. **Obi L. Griffith:** writing – review and editing, methodology, software, validation, formal analysis, data curation, supervision, funding acquisition. **Vikas Prasad:** writing – review and editing, visualization. **Mariam Khanfar:** writing – review and editing, formal analysis, data curation, visualization. **Malachi Griffith:** writing – review and editing, methodology, validation, software, formal analysis, data curation, supervision. **Paul Simonson:** writing – review and editing, methodology, software, validation, formal analysis, data curation, supervision. **Thomas Odeny:** conceptualization, writing – review and editing, supervision. **Ethel Cesarman:** writing – review and editing, supervision. **Lee Ratner:** conceptualization, writing – original draft, writing – review and editing, data curation, supervision, project administration, funding acquisition. **Jeanne Jiang:** writing – review and editing, methodology.

## Funding

The study was funded by R21 CA‐257493, supplemental funding award P30 CA‐09184‐19S2, and an AIDS Malignancy Consortium Scholar Award, UM1 CA121947.

## Ethics Statement

The study compiled with ethical standards outlined in the Belmont Report and was approved by the Washington University Institutional Human Studies Review Committee (approval number 201104376).

## Conflicts of Interest

The authors declare no conflicts of interest.

## Supporting information


**Figure S1:** Trend in KSHV viral load before and throughout treatment.
**Figure S2:** Percentage of KSHV+ and CD38+ cells per KS lesion.
**Figure S3:** B cell clonotype abundance in the baseline PEL and complete remission PBMC samples, CD38 and CD45 expression per cell in each PBMC sample, and cell identity distribution among cells displaying a PEL gene signature.

**Data S1:** Supervised gating workflow for multiplex immunofluorescence study shown in Figure 4.

## Data Availability

The data that support the findings of this study are openly available in dbGaP at https://dbgap.ncbi.nlm.nih.gov/home/, reference number phs003800.v2.p1.
