# Supplementary material for: Case Report: Synchronous Manifestations of Kaposi Sarcoma Herpesvirus‐Associated Disorders
Source: Cancer Rep (Hoboken). 2026 Jun 1;9(6):e70589. doi: 10.1002/cnr2.70589 (PMC13238725; doi:10.1002/cnr2.70589)
Supplement: Supplementary file 1 — Figure S1: Trend in KSHV viral load before and throughout treatment. Figure S2: Percentage of KSHV+ and CD38+ cells per KS lesion. Figure S3: B cell clonotype abundance in the baseline PEL and complete remission PBMC samples, CD38 and CD45 expression per cell in each PBMC sample, and cell identity distribution among cells displaying a PEL gene signature. [file CNR2-9-e70589-s001.pdf]

Supplemental Figure Legends

Supplemental Figure 1: This chart demonstrates the trend in KSHV viral load before and throughout treatment. The red line indicates the start of DA-R-EPOCH. The blue line indicates the single cycle of salvage gemcitabine and oxaliplatin prior to disease progression. The green line indicates the start of daratumumab. The patient began experiencing a significant decline in KSHV viral load following the initiation of DA-R-EPOCH that continued despite disease progression. His viral load became undetectable while on treatment with daratumumab.

Supplemental Figure 2: A) Percentage of KSHV+ cells per KS lesion. KSHV+ cells were determined as having at least 2 UMIs from any KSHV genes (shown in blue). The orange bars show cells featuring only 1 UMI from any KSHV gene. B) CD38+ cells per skin sample, shown as a percentage of the total lesion cells for each sample. Of note, KS7.1 and KS7.2 are duplicates of the same lesion from subject, KS7.

Supplemental Figure 3: A) B cell clonotype abundance in the baseline PEL (KS14A) and complete remission (KS14B) PBMC samples. The barcode frequency represents the number of individual B cells assigned to each unique B-cell receptor (BCR) clonotype, based on their reconstructed V(D)J sequences sequences from multi-omic analysis of scRNAseq using 10X genomics BCRseq. The kappa lambda ratio is approximately 2 for both samples. B) Violin plot representing the distribution of CD45 expression per cell in each PBMC sample. The expression of CD45 (PTPRC) is normalized to the total UMI count per cell in each sample and shown in logarithmic scale. For the KS14 samples, ‘erythroid’ indicates cells classified by SingleR as bone marrow-derived, erythroblasts, or pro-myelocytes. ‘PBMC’ indicates all other cells in the sample. C) Cell identity distribution among cells displaying a PEL gene signature (CD20-, CD19-, CD79a-, PAX5-) and CD45>4 transcripts across all PBMC samples. D) Percentage of total CD38+ cells in each PBMC sample excluding erythrocytes, bone marrow-derived cells, and pro-myelocytes from samples KS14A and KS14B. The aforementioned cell types are either absent or constitute a negligible population in all other PBMC samples. CD38+ cells were included by featuring at least 1 CD38 transcript. Proportions in this table are comparable to those shown in Figure 3G, which includes all cell types.

Fig S1

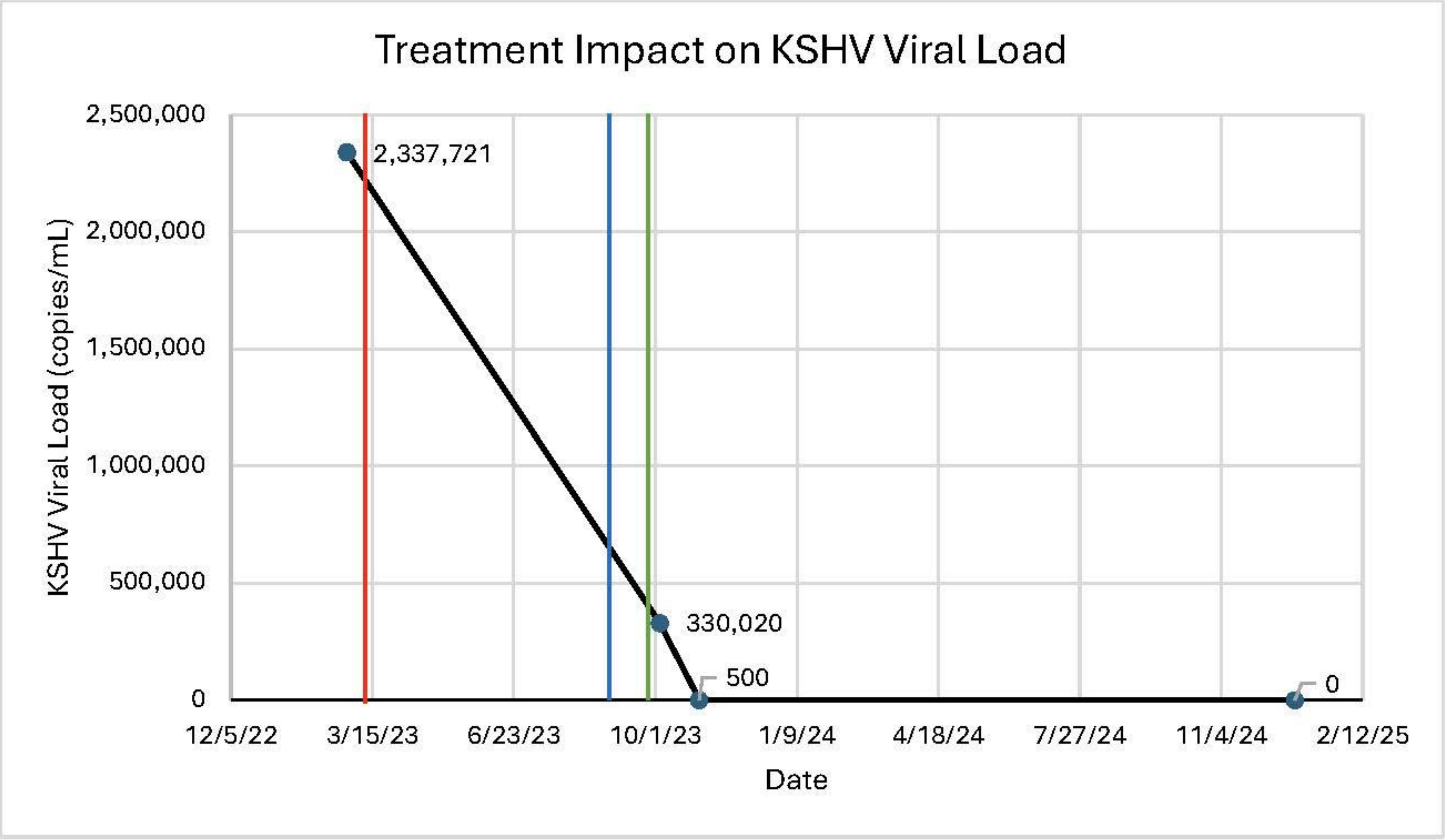
Treatment Impact on KSHV Viral Load
KSHV Viral Load (copies/mL)
2,500,000
2,000,000
1,500,000
1,000,000
500,000
0
2,337,721
330,020
500
0
12/5/22
3/15/23
6/23/23
10/1/23
1/9/24
4/18/24
7/27/24
11/4/24
2/12/25
Date

Fig S2

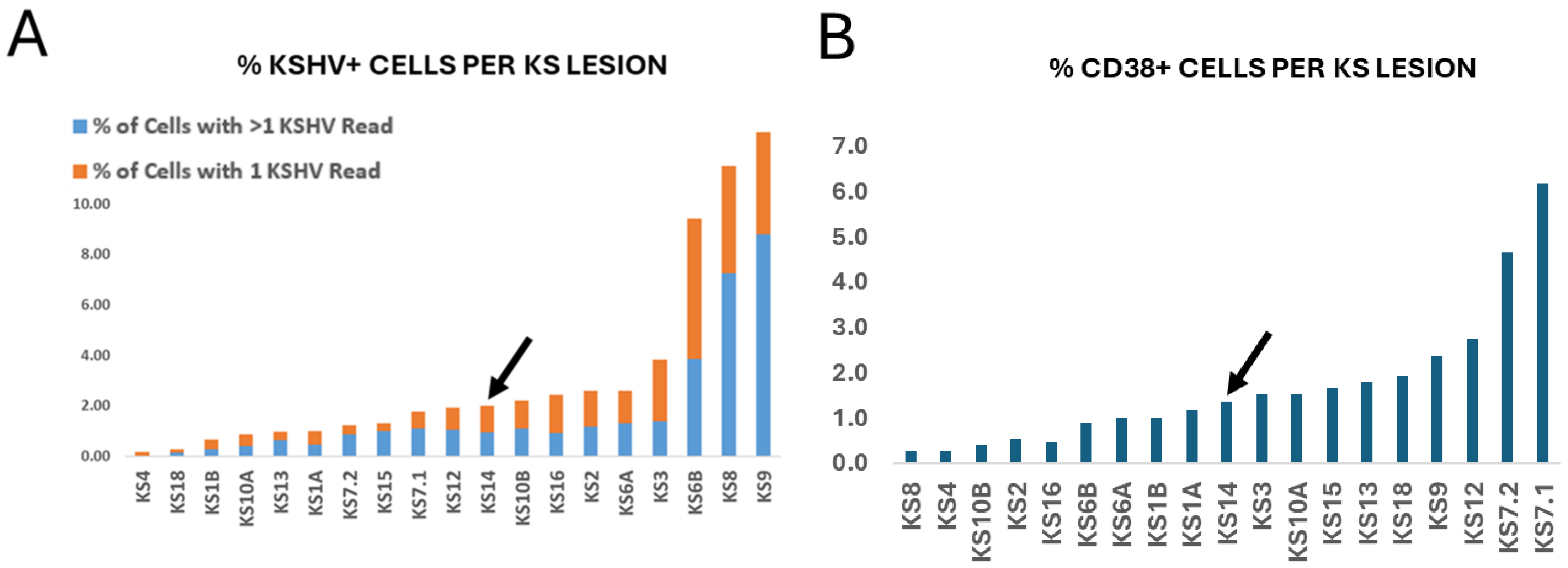
A
% KSHV+ CELLS PER KS LESION
% of Cells with >1 KSHV Read
% of Cells with 1 KSHV Read
10.00
8.00
6.00
4.00
2.00
0.00
KS4
KS18
KS1B
KS10A
KS13
KS1A
KS7.2
KS15
KS7.1
KS12
KS14
KS10B
KS16
KS2
KS6A
KS3
KS6B
KS8
KS9
B
% CD38+ CELLS PER KS LESION
7.0
6.0
5.0
4.0
3.0
2.0
1.0
0.0
KS8
KS4
KS10B
KS2
KS16
KS6B
KS6A
KS1B
KS1A
KS14
KS3
KS10A
KS15
KS13
KS18
KS9
KS12
KS7.2
KS7.1

Fig S3

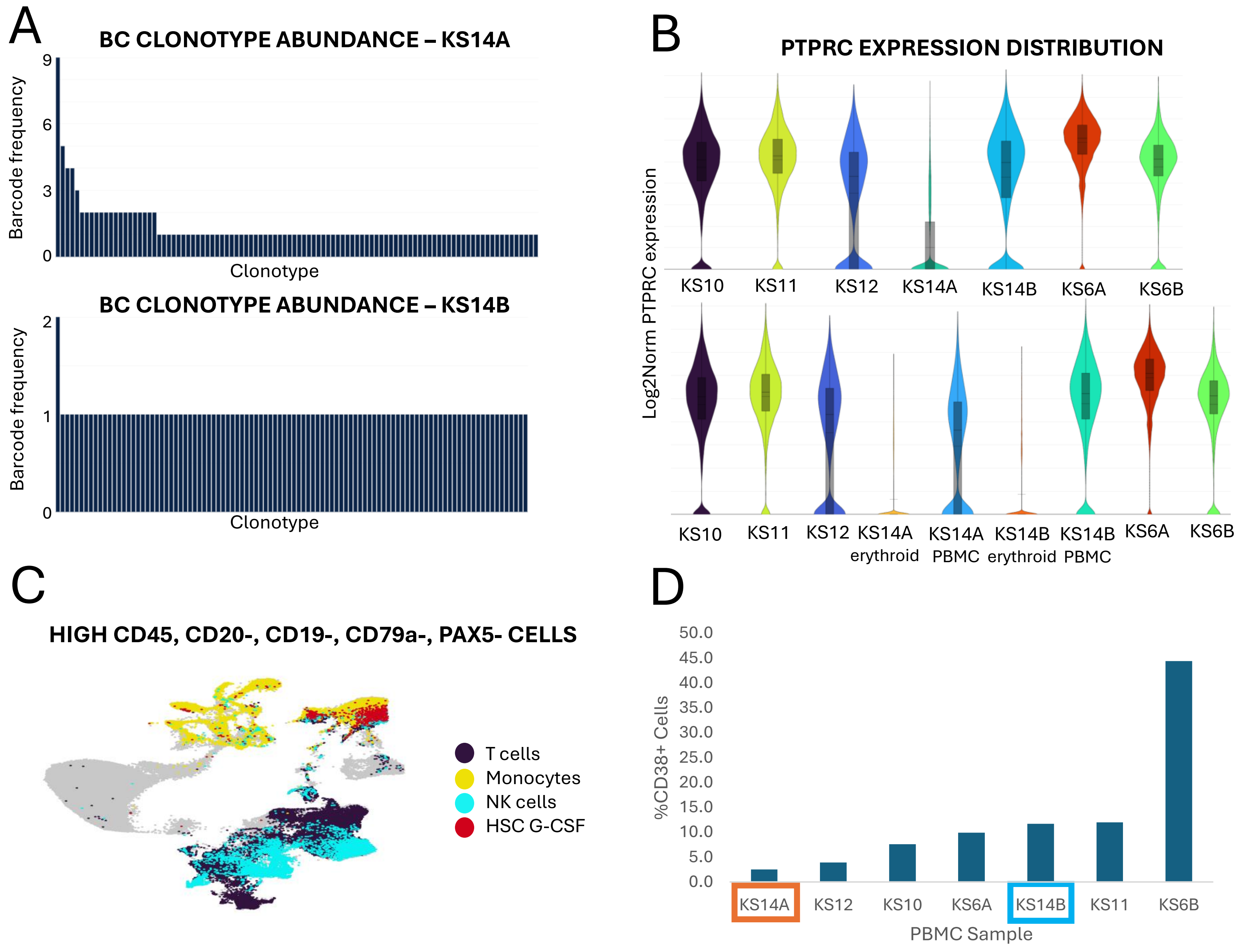
A
BC CLONOTYPE ABUNDANCE – KS14A
Barcode frequency
9
6
3
0
Clonotype
BC CLONOTYPE ABUNDANCE – KS14B
Barcode frequency
2
1
0
Clonotype
B
PTPRC EXPRESSION DISTRIBUTION
Log2Norm PTPRC expression
KS10
KS11
KS12
KS14A
KS14B
KS6A
KS6B
KS10
KS11
KS12
KS14A erythroid
KS14A PBMC
KS14B erythroid
KS14B PBMC
KS6A
KS6B
C
HIGH CD45, CD20-, CD19-, CD79a-, PAX5- CELLS
T cells
Monocytes
NK cells
HSC G-CSF
D
%CD38+ Cells
50.0
45.0
40.0
35.0
30.0
25.0
20.0
15.0
10.0
5.0
0.0
KS14A
KS12
KS10
KS6A
KS14B
KS11
KS6B
PBMC Sample
